# Perceived antimicrobial dispensing practices in medicine outlets in Ghana: A maximum difference experiment design

**DOI:** 10.1371/journal.pone.0288519

**Published:** 2023-07-13

**Authors:** Eric Nyarko, Francisca Mawulawoe Akoto, Kwabena Doku-Amponsah

**Affiliations:** Department of Statistics and Actuarial Science, School of Physical and Mathematical Sciences, University of Ghana, Legon, Accra, Ghana; The University of Jordan School of Pharmacy, JORDAN

## Abstract

**Introduction:**

Antimicrobials are consumed among patients globally, but in developing and middle-income countries, these drugs can be obtained without a prescription from pharmacies and licensed drug stores due to inadequate regulation in the pharmaceutical sector. This study aimed to assess antimicrobial dispensing practices in medicine sales outlets (i.e., pharmacies and licensed drug stores) to provide quantitative evidence for policy discussions to enhance patient safety and care quality in Ghana’s pharmaceutical industry.

**Method:**

The data for this study were obtained from a cross-sectional survey conducted in the Greater Accra region between July and August 2022. The survey was conducted through interviewer-administered questionnaires, and 200 staff members from medicine sales outlets were randomly selected using a two-stage cluster and random sampling technique. The maximum difference experiment model, rooted in random utility theory, was used to analyze their antimicrobial dispensing practices.

**Result:**

We found that medicine sales outlet staffs were highly concerned about following the drug act and not dispensing antimicrobials without a prescription, and usually refer a patient to get a prescription from a doctor if the patient has complications (like high fever, generalized malaise, fatigue as symptoms, sinusitis). Stronger concerns were also observed for medicine outlet staff not dispensing antimicrobials without a prescription if the patient is pediatric or geriatric with a severe infection. They also evaluated patients and dispensed antimicrobials based on symptoms, not their age or gender. However, they tended not to dispense antibiotics if the patient had a mild fever and requested it without a prescription.

**Conclusion:**

Our results provide insight into the need for a national surveillance system for monitoring antimicrobial prescribing and dispensing practices at medicine sales outlets. Therefore, the selection of antimicrobials for treating infectious diseases may be informed by evidence-based antimicrobial prescribing and dispensing surveillance data and will help policymakers to know the pattern of commonly consumed antimicrobials in the medicine sales outlets.

## Introduction

It is a common practice for patients around the world to use antimicrobial agents, both with and without a prescription [1,2]. Research shows that in low- and middle-income countries, approximately two-thirds of the antibiotics purchased in pharmacies are consumed without a prescription, and 20–50% of those are misused when treating bacterial infections [3,4]. These countries typically have easy access to antimicrobials, which can be obtained without a prescription from medicine sales outlets including pharmacies and licensed drug stores. Unfortunately, these pharmaceutical sectors are poorly regulated [5,6], leading to inadequate supervision, control, prescribing, and dispensing processes. This results in poor compliance with antibiotic dispensing guidelines in developing and middle-income nations.

According to findings, antibiotics are among Africa’s most frequently prescribed medications. It was discovered that 90% of individuals with acute illnesses seek medical care outside their homes, and 95% receive medication, with 36% being antibiotics. Most of these antibiotics (75%) were Metronidazole, Cotrimoxazole, and Amoxicillin. Shockingly, one in four people sought antibiotics from unofficial sources, and over 30% did so without a prescription [7]. In Tanzania, many medications were supplied without a prescription and were either requested by customers or suggested by pharmacists. Furthermore, dosage instructions were rarely given by staff at medicine sales outlets, and when provided, the information was often inconsistent with recommendations [8].

In Nairobi, a study found that 64% of retail pharmacies sold medications without a prescription. Many of these stores also provided lower doses of antibiotics, particularly outside the city center. The study also discovered that while 31% of guardians received antibiotics, 38% were not prescribed by a doctor [9]. A study in Uganda’s Mukono district found that, 93.5% of drug stores distributed antibiotics, including Amoxicillin and Trimethoprim-Sulfamethoxazole. The study also revealed that overprescription was linked to the dispenser’s professional background. Another study revealed that, despite oral rehydration salts and zinc pills being recommended as the first line of treatment for children with diarrhea, 29.4% of pharmacy staff members still chose antibiotics as their first option. Only 8.2% of providers had received antibiotic training, while 10.6% had received training in managing pneumonia cases [10].

A recent study in Northwest Cameroon analyzed the prescribing trends and factors influencing antibiotic prescription in primary healthcare facilities. Results showed that 36.71% of patients received antibiotics, with an average of 1.14 prescribed antibiotics per patient. Amoxicillin was the most commonly prescribed antibiotic, accounting for 29.9% of prescriptions, and respiratory tract infections were the primary reason for 21.27% of prescriptions. Nearly all prescriptions (99.87%) were from the Essential Drug List and prescribed by generic name (98.36%). The study found that laboratory results, patient involvement, and performance-based financing strongly influenced the prescribing rates of antibiotics [11]. Additionally, a separate study discovered that before law enforcement, 70.7% of dispensers sold antibiotics without a prescription. In comparison, 96.6% and 87.7% of dispensers sold antibiotics without a prescription for pharyngitis and urinary tract infections, respectively [12].

In Ghana, the Health Professions Regulatory Act, 2013 (Act 857) only allows medical professionals like medical doctors, physician assistants, midwives, and nurses who have received prescribing training to write registered antibiotic prescriptions [13]. The Pharmacy Act of 1994 (Act 489) also includes guidelines for the dispensing and selling of drugs, including antibiotics. Pharmacies may dispense certain antibiotics like Amoxicillin, Flucloxacillin, Norfloxacin + Tinidazole, Ciprofloxacin, Doxycycline, Tetracycline, Erythromycin, and Ampicillin based on the recommendation of a practicing pharmacist. However, licensed chemical sellers are limited to selling only Cotrimoxazole for treating specific infections like infective diarrhea, urinary tract infections, and upper respiratory tract infections. Moreover, if a restricted drug is prescribed, the supplier must enter the date and information on the prescription and retain it for two years in a manner as to be readily available for inspection [13,14].

Although the Pharmacy Act exists in Ghana, antimicrobial agents are easily accessible in medicine sales outlets, as in many other developing and middle-income countries. These outlets are often the first point of contact for ill patients [1]. Here the phrase "outlets" refers to both pharmacies and licensed drug stores. Medicine sales outlets are registered with the Pharmacy Council of Ghana [15], to offer pharmaceutical services. However, little is known about the prescribing and dispensing of antimicrobials in these outlets, where most sick individuals seek treatment. Misuse of antimicrobial drugs can lead to the emergence, dissemination, and persistence of resistant microorganisms, which poses a global hazard [7]. Therefore, governments are concerned about the excessive use of antimicrobials and the increasing prevalence of bad prescribing and dispensing practices due to the implications of antimicrobial resistance habits [3,7].

Few recent studies have investigated the practices of prescribing and dispensing antimicrobial drugs in medicine outlets in Ghana [16–21]. However, they do not model or provide a quantification of these practices. Our study aims to fill this knowledge gap by conducting an in-depth analysis of the antimicrobial dispensing practices in medicine sales outlets (so-called pharmacies and licensed drug stores) in Accra, Ghana. We utilized a best-worst scaling (BWS) experiment (also known as maximum difference scaling) to quantify medicine sales outlet staff’s antimicrobial dispensing practices. This research is crucial as it can help improve patient safety and care quality in Ghana’s pharmaceutical industry by identifying areas that require interventions to enhance antimicrobial sales in medicine outlets. The results of this study could serve as a valuable guide for discussions and decision-making in the pharmaceutical sector in Ghana and other developing countries.

## Methodology

### Study area

The study was conducted in the Greater Accra region, bordered by the Eastern region to the north, the Volta Region to the east, the Gulf of Guinea to the south, and the Central region to the west. As the capital city of Ghana, the Greater Accra region is the country’s smallest region, covering an area of 3,245 km^2^, which is 1.4% of Ghana’s total geographical area. It has 16 administrative districts and is the second most populous area after the Ashanti Region, with a population of 5,455,692 people in 2021, which is 17.7% of Ghana’s total population. A high proportion, 87.4% of its population, resides in urban areas [22].

### Sample size and data collection

To conduct this study, we surveyed staff from 200 medicine sales outlets in Greater Accra between July and August 2022. We used a two-stage cluster and random sampling technique to obtain the data collected through interviewer-questionnaire administration. The minimum permissible sample size for simple random sampling depends on the required degree of accuracy of computed probabilities. Let *g* denote the true choice proportion of the study population, *a* be the allowable deviation as a percentage between $\hat{g}$ and *g*, *q* = 1−*g*, α be the level of confidence in the estimations such that $Pr(|\hat{g}-g|\leq ag)\geq \alpha$ for any given sample *n* [23] under the standard normal distribution is given by

$$n\geq \frac{q}{ga^{2}}{\left[\Phi^{-1}(1-\frac{\alpha }{2})\right]}^{2}.$$

The estimated sample size desired for this study under the assumption of equal choice probabilities (i.e., *g* = 50%) is given by

$$n\geq \frac{0.5}{0.5\times 0.05^{2}}{\left[1.96\right]}^{2}$$

n≥1536.

A minimum of 192 respondents were needed based on the number of available BWS scenarios (*k =* 8) and the formula *N = n/k*. The anticipated sample size of 200 respondents was sufficient, and all 200 were included in the final analysis due to a 100% response rate. Prior to data collection, written consent was obtained from respondents after explaining the study’s purpose and emphasizing their voluntary participation. Respondents who had dispensed antimicrobials within the past three months to one year and provided informed consent were included in the study. However, those who refused consent and five interns in four medicine sales outlets who had never dispensed antimicrobials over the past three months and lacked experience were excluded from this study. Respondents were compensated with airtime for their time spent answering questionnaires.

### Experimental design

Best-wort scaling (BWS) is a contemporary approach for conducting choice experiments [24] that have gained popularity in various fields such as health, conflict handling, environmental sustainability, and food-related consumer research [25–29]. This method enables the evaluation of the relative importance of attributes on a common scale [30] , which is crucial for this study. In BWS, participants select their most preferred and disliked items (i.e., specific characteristics) among three or more options. To identify the factors related to antimicrobial prescribing and dispensing practices, we consulted medical sales experts and conducted an extensive literature review [31]. The initial list of factors was then narrowed to eight plausible attributes through focus group discussions with eight medicine sales outlet staff. These eight attributes are presented in Table 1.

**Table 1:** Items considered in the BWS questionnaire.

	Attributes
1	Follow the drug act and do not dispense antimicrobials without a prescription
2	Dispense antibiotics if the patient has a good outcome with previously prescribed antibiotic
3	Dispense antibiotics to poor patients who cannot afford doctor/hospital charges keeping in mind their cost-effectiveness
4	Evaluate patients and dispense antimicrobials based on symptoms, and not their age or gender
5	Dispense antimicrobials when a patient requests them without a prescription
6	Dispense antimicrobials if the patient has a mild fever
7	Do not dispense antimicrobials and usually refer the patient to get a prescription from a doctor if a patent has a high fever, generalized malaise, fatigue as symptoms, sinusitis
8	Do not dispense antimicrobials without a prescription for a pediatric or geriatric patient with a severe infection

The eight plausible attributes of antimicrobial dispensing practices were obtained through focus group discussions with eight medicine sales outlet staff.

**Fig 1:**
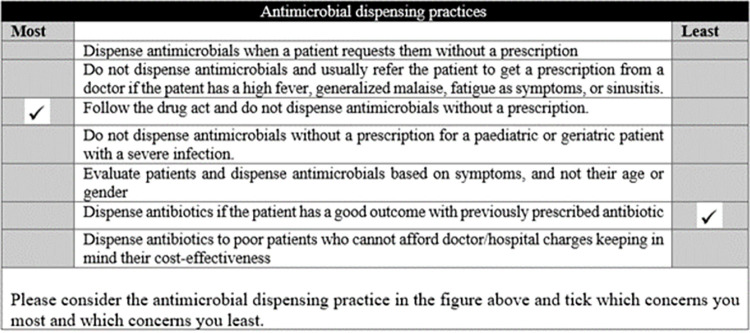
A sample completed best-worst scaling scenario. The eight sets of best-worst scaling scenarios generated from the balanced incomplete block design contain seven attributes each. The sample completed best-worst scaling scenario represents best-worst scaling scenario number 1 in the balanced incomplete block design and contains plausible attributes 5, 7, 1, 8, 4, 2, and 3.

At the start of the questionnaire, we collected data about the participants’ demographics and asked questions about their practices regarding prescribing and dispensing antimicrobial medications. We then presented respondents with eight sets of BWS scenarios, each with seven attributes related to antimicrobial dispensing practices. For each set, we asked participants to indicate which attribute of antimicrobial dispensing practices concerned them the most and least. Each set of the BWS scenarios was presented separately. The balanced incomplete block design [32] was used to allocate the eight plausible attributes in the BWS scenarios. Fig 1 shows a completed example best-worst scaling scenario.

#### Ethical considerations

All methods were carried out following relevant guidelines and regulations. Ethical clearance was obtained from the University of Ghana Ethical and Protocol Review Committee (Reference No: ECBAS 034/21-22). Before collecting data, permission was obtained in the form of written consent from the respondents after explaining the purpose of the study. Respondents were informed that participation was voluntary and they could choose whether to participate in the study. Extra precautions were taken due to the COVID-19 outbreak, such as wearing face masks and providing hand-cleansing sanitizers, soaps, and water before interacting with the study participants. We followed all COVID-19 preventive protocols during each personal interaction and data collection session.

### Empirical strategy

In BWS, items are evaluated based on a random utility framework [33–35], and the frequency of choices made by participants serve as a metric to compare the relative importance of different items. By using a model with an error theory, it is possible to estimate how often one item might be chosen over another. Here a choice is assumed to have an underlying value, or utility, to respondents. The maximum difference model is used to estimate these utilities. To formalize this model, let ℏ with |ℏ|≥3 denote the finite set of potentially available choice options in a given best-worst scenario and let *ψ*(ℏ) denote the experimental design, that is, the set of (sub)sets of antimicrobial dispensing choice alternatives that occur in this study. For any set *Y*∈*ψ*(ℏ), *Y*⊆ℏ with |*Y*|≥3, let $M_{Y}(x)$ denote the probability that the alternative *x* is chosen as best in set *Y*, $A_{Y}(y)$ the probability that the alternative *y* is chosen as worst in set *Y*, and $MA_{Y}(x,y)$ the probability that alternative *x* is chosen as best in set *Y* and the alternative *y*≠*x* is chosen as worst in *Y*.

Now, the best choice model assumes that there is a scale μ such that for all *y*∈*Y*∈*ψ*(ℏ),

$$M_{Y}(y)=\frac{e^{\mu (y)}}{\sum_{z\in Y}e^{\mu (z)}},$$ (1)

where the value μ(*y*) for an option *y* is interpreted as the utility for that option. The parallel model for worst choice assumes that there is a scale *v* such that for all *y*∈*Y*∈*ψ*(ℏ),

$$A_{Y}(y)=\frac{e^{\nu (y)}}{\sum_{z\in Y}e^{\nu (z)}}.$$ (2)

Assuming that (1) and (2) are met and that the choice probabilities for sets of two elements follow a plausible condition for all different pairs, *x*,*y*∈*Y*∈*ψ*(ℏ), then

$$M_{\left\{x,y\right\}}(x)=A_{\left\{x,y\right\}}(y),$$

that is, we have

$$A_{Y}(y)=\frac{e^{-\mu (y)}}{\sum_{z\in Y}e^{-\mu (z)}}.$$ (3)

If the choice probabilities that are considered the best (and worst) satisfy (1) and (3), then when selecting the best option, the utility of a choice alternative is the opposite of the utility of that same option when selecting the worst option, and this utility-scale μ is such that for all *x*,*y*∈*Y*∈*ψ*(ℏ), *x*≠*y*,

$$MA_{Y}(x,y)=\frac{e^{\left[\mu (x)-\mu (y)\right]}}{\sum_{\{p,q\}\in Y}e^{\left[\mu (p)-\mu (q)\right]}}.$$ (4)

Here, a respondent chooses the items they like and dislike the most. This data is analyzed using the maximum difference model, treating all attributes as generic variables. Statistical analysis was conducted using John’s Macintosh Project Pro (JMP Pro Version 16.0). The significance of the attributes determining antimicrobial dispensing practices was assessed at the 95% confidence intervals (CIs). The relative importance of the attributes is compared through parameter estimates. A parameter estimate is considered statistically significant when the associated 95% confidence interval (CI) is greater than or less than zero. If the 95% CI overlaps, the parameter estimate is not significantly different. A significant positive/negative parameter estimate indicates a high/low preference for a specific attribute.

## Results

### Demographic description

This study included 200 staff members from medicine sales outlets, all of whom responded. Table 2 provides an overview of the population studied. Of the 200 respondents, 106 (77.4%) were male and aged between 18 and 30 years old, while 47 (74.6%) were female. Most of the respondents, 123 (89.8%), were male Christians who had obtained a first degree 85 (62.0%), lived alone 119 (86.9%), and had no children 119 (86.9%). Among the female respondents, the majority were female Christians who had obtained a first degree 30 (47.6%), lived alone 42 (66.7%), and had no children 49 (77.8%). More than half 116 (84.7%) of the male respondents had 1–5 years of working experience, with 127 (92.7%) working in pharmacy stores as pharmacists 65 (47.4%). Among the female respondents, 50 (79.4%) had 1–5 years of working experience, with 58 (92.1%) working in pharmacy stores and 30 (47.6%) as pharmacists. 54 (39.4%) male respondents had participated in an antimicrobial dispensing training program organized by the pharmacy council, and 49 (35.8%) had training from the professional staff at their facility or manager. In comparison, 24 (38.1%) of the female respondents had participated in an antimicrobial training program organized by the pharmacy council, and 23 (36.5%) had training from the professional staff at their facility or manager.

**Table 2:** Demographic description of the study population.

Variable	Gender Female Male		
**Age (years)**			
	18–30	47 (74.6%)	106 (77.4%)
	31–45	16 (25.4%)	28 (20.4%)
	46–60	0 (0.0%)	3 (2.2%)
**Educational level completed**	Secondary School/SHS/SSS or less	14 (22.2%)	20 (14.6%)
	Diploma/HND	15 (23.8%)	28 (20.4%)
	Undergraduate	30 (47.6%)	85 (62.0%)
	Post-graduate degree	4 (6.3%)	4 (2.9%)
**Religion**	Christian	49 (77.8%)	123 (89.8%)
	Muslim	14 (22.2%)	11 (8.0%)
	Traditional	0 (0.0%)	3 (2.2%)
**Marital status**	Living alone	42 (66.7%)	119 (86.9%)
	Living with another (married, partnership)	21 (33.3%)	18 (13.1%)
**Number of children**	None	49 (77.8%)	119 (86.9%)
	1–3	13 (20.6%)	13 (9.5%)
	4–6	1 (1.6%)	0 (0.0%)
	>6	0 (0.0%)	5 (3.6%)
**Type of facility you work**	Drug store	5 (7.9%)	10 (7.3%)
	Pharmacy store	58 (92.1%)	127 (92.7%)
**Current job role**	License chemical seller	3 (4.8%)	4 (2.9%)
	Pharmacist	30 (47.6%)	65 (47.4%)
	Pharmacy technician	11 (17.5%)	46 (33.6%)
	Pharmacy/chemical shop assistant	19 (30.2%)	22 (16.1%)
**Length of work experience (years)**	1–5	50 (79.4%)	116 (84.7%)
	>5	13 (20.6%)	21 (15.3%)
**Institution you have been involved in antimicrobial training programme**	Ghana health service	9 (14.3%)	12 (8.8%)
	Pharmaceutical companies	7 (11.1%)	22 (16.1%)
	Pharmacy council	24 (38.1)	54 (39.4%)
	Trained by a professional staff at my facility/manager	23 (36.5%)	49 (35.8%)

Out of the 200 staff members sampled from medicine sales outlets, a significant number were men aged between 18 and 30 years old. These individuals were graduate pharmacists with 1–5 years of work experience and were employed in pharmacy stores. They also participated in multiple antimicrobial training programs organized by the pharmacy council.

### Maximum difference model results

**Fig 2:**
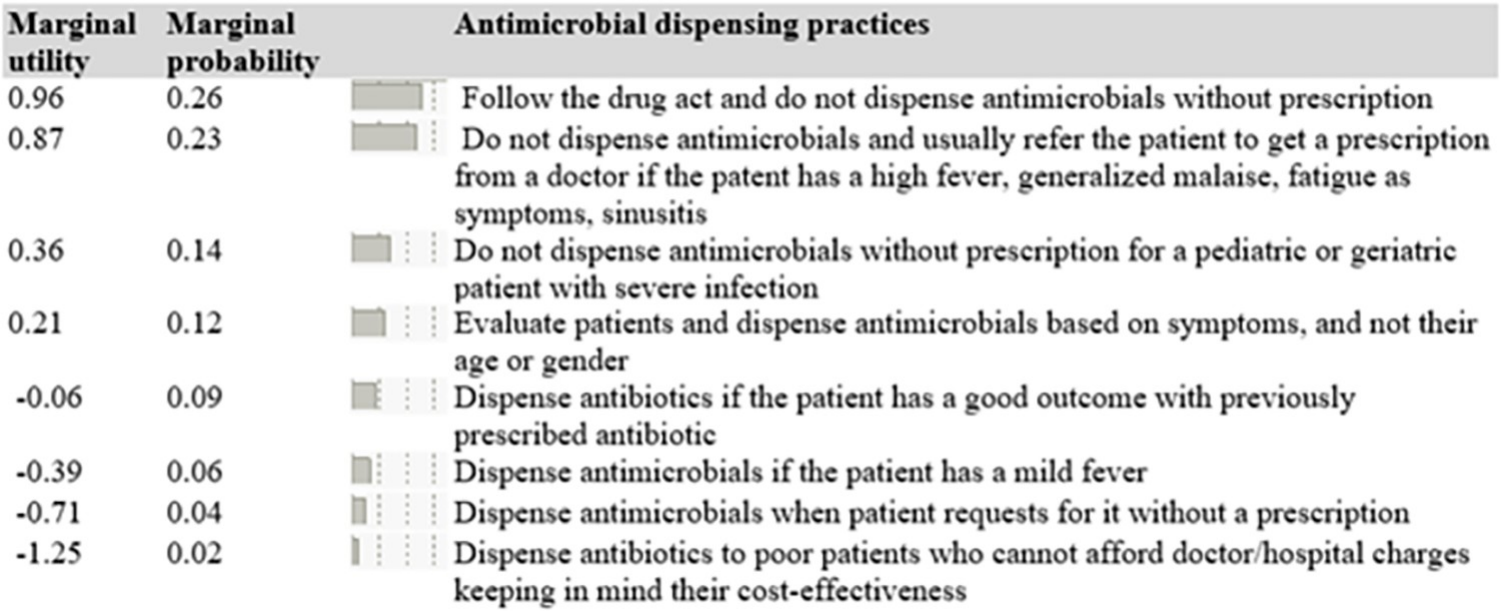
Marginal utility estimates and marginal probability of attributes associated with antimicrobial dispensing practices. The corresponding estimates of marginal utility and probability values determine the importance attached to each attribute. The length of the bars represents these values.

The results of our model on antimicrobial dispensing practices, including the utility coefficients (also-called parameter or utility estimates), standard errors (SE), and 95% confidence intervals (CIs) are presented in Table 3. The statistical significance of the model was measured at *p*-value (*p*< 0.00, 0.01 and 0.05). The likelihood ratio (L-R) Chi-square value of 561.46 with 7 degrees of freedom (DF) and a *p*< 0.00 tell us that the model as a whole is important at a pre-specified significance level of 1%, 5% and 10%. To determine the statistical significance of a utility coefficient, we check if the 95% CIs are greater than or less than zero. If there is an overlap of the 95% CIs, the utility coefficient associated with the corresponding attribute is not significantly different. All parameter estimates of attributes related to antimicrobial dispensing practices have the expected sign at 95% CIs (Table 3). The relative importance of each attribute is shown in Fig 2 with their corresponding marginal utility estimates, and marginal probability values (represented by the length of the bars).

**Table 3:** Maximum difference model results of attributes associated with antimicrobial dispensing practices.

Attribute	Parameter Estimate	SE	Lower 95%	Upper 95%
Dispense antimicrobials if a patient has a mild fever	-0.39	0.08	-0.55	-0.23
Dispense antimicrobials when patient requests for it without a prescription	-0.71	0.07	-0.86	-0.55
Do not dispense antimicrobials and usually refer a patient to get a prescription from a doctor if the patient has a high fever, generalized malaise, fatigue as symptoms, sinusitis	0.87	0.07	0.72	1.02
Follow the drug act and do not dispense antimicrobials without prescription	0.96	0.07	0.81	1.11
Do not dispense antimicrobials without prescription for a pediatric or geriatric patient with severe infection	0.36	0.08	0.20	0.52
Evaluate patients and dispense antimicrobials based on symptoms, and not their age or gender	0.21	0.08	0.05	0.37
Give antibiotics if the patient has a good outcome with previously prescribed antibiotic	-0.06	0.08	-0.22	0.09
**Model fits**				
L-R Chi-Square	561.46			
AIC	3915.50			
BIC	3946.06			
DF	7			
*P-Value*	<0.00			

*SE, standard error; L-R, likelihood ratio; AIC, Akaike information criteria; BIC, Bayesian information criteria; DF, degree of freedom. The attribute variable following the drug act and not dispensing antimicrobials without a prescription has the highest utility. Other stronger preferences are observed for not dispensing antimicrobials and usually referring a patient to get a prescription from a doctor if the patient has complications (like high fever, generalized malaise, fatigue as symptoms, sinusitis), followed by not dispensing antimicrobials without a prescription if a patient is pediatric or geriatric with a severe infection as well as evaluate patients and dispense antimicrobials based on symptoms, not their age or gender. However, the attribute variables, such as dispensing antibiotics if a patient has a mild fever and requests an antimicrobial without a prescription, result in disutility.

It can be inferred that, relatively, respondents follow the drug act and avoid dispensing antimicrobials without a prescription (marginal utility estimate = 0.96 with a marginal probability value = 0.26). The results showed stronger preferences for not dispensing antimicrobials and referring patients to a doctor if they have symptoms like high fever, fatigue, and sinusitis (marginal utility estimate = 0.87 with a marginal probability value = 0.23). Additionally, respondents were less likely to dispense antimicrobials without a prescription for pediatric or geriatric patients with severe infections (marginal utility estimate = 0.36 with a marginal probability value = 0.14). Respondents prioritize evaluating patients based on their symptoms when dispensing antimicrobials rather than their age or gender (marginal utility estimate = 0.21 with a marginal probability value = 0.12). On the other hand, there was a disutility associated with dispensing antimicrobials if the patient has a mild fever (marginal utility estimate = -0.39 with a marginal probability value = 0.06), as well as dispensing antimicrobials when a patient requests it without a prescription (marginal utility estimate = -0.71 with marginal probability value = 0.04). Overall, the survey suggested that medicine sales staffs were careful when dispensing antimicrobials.

Table 4 compares antimicrobial dispensing practices and their associated attributes, including utility estimates, marginal probabilities, and odd ratios. Patients with mild fevers have a higher chance of receiving antimicrobials (odds ratio = 1.37; probability = 0.57) than those who request them without a prescription (odds ratio = 0.72; probability = 0.42). Overall, medicine sales outlet staff are more likely to follow the drug act by not dispensing antimicrobials without a prescription (odds ratio = 3.89; probability = 0.79) and referring patients with high fevers, malaise, fatigue, and sinusitis to a doctor for a prescription (odds ratio = 3.55; probability = 0.78). Dispensing antimicrobials to pediatric or geriatric patients with severe infections without a prescription has higher odds (odds ratio = 2.12; probability = 0.68), and medicine sales outlet staff are more likely to evaluate patients based on symptoms, not age or gender, when dispensing antimicrobials (odds ratio = 1.83; probability = 0.64). Again, dispensing antimicrobials without a prescription when a patient requests them has lower odds (odds ratio within the range 0.18–0.39; probability within the range 0.15–0.28) than following the drug act (odds ratio = 5.37; probability = 0.84), referring pediatric or geriatric patients with severe infections to a doctor for a prescription (odds ratio = 2.93; probability = 0.74), and evaluating patients based on symptoms when dispensing antimicrobials (odds ratio = 2.52; probability = 0.71).

**Table 4:** All levels comparison of attributes associated with antimicrobial dispensing practices.

Compared1	Compared2	Utility1	Utility2	Probability1	Probability2	Odds1	Odds2
Dispense antimicrobials if the patient has a mild fever	Dispense antimicrobials when patient requests for it without a prescription	-0.39	-0.71	0.57	0.42	1.37	0.72
	Do not dispense antimicrobials and usually refer the patient to get a prescription from a doctor if the patent has a high fever, generalized malaise, fatigue as symptoms, sinusitis	-0.39	0.87	0.21	0.78	0.28	3.55
	Follow the drug act and do not dispense antimicrobials without prescription	-0.39	0.96	0.20	0.79	0.25	3.89
	Do not dispense antimicrobials without prescription for a pediatric or geriatric patient with severe infection	-0.39	0.36	0.31	0.68	0.46	2.12
	Evaluate patients and dispense antimicrobials based on symptoms, and not their age or gender	-0.39	0.21	0.35	0.64	0.54	1.83
	Dispense antibiotics if the patient has a good outcome with previously prescribed antibiotic	-0.39	-0.06	0.41	0.58	0.72	1.38
Dispense antimicrobials when patient requests for it without a prescription	Follow the drug act and do not dispense antimicrobials without prescription	-0.71	0.96	0.15	0.84	0.18	5.37
	Do not dispense antimicrobials without prescription for a pediatric or geriatric patient with severe infection	-0.71	0.36	0.25	0.74	0.34	2.93
	Evaluate patients and dispense antimicrobials based on symptoms, and not their age or gender	-0.71	0.21	0.28	0.71	0.39	2.52
Do not dispense antimicrobials and usually refer the patient to get a prescription from a doctor if the patent has a high fever, generalized malaise, fatigue as symptoms, sinusitis	Dispense antimicrobials when patient requests for it without a prescription	0.87	-0.71	0.83	0.16	4.89	0.20
	Follow the drug act and do not dispense antimicrobials without prescription	0.87	0.96	0.47	0.52	0.91	1.09
	Do not dispense antimicrobials without prescription for a pediatric or geriatric patient with severe infection	0.87	0.36	0.62	0.37	1.66	0.59
	Give antibiotics if the patient has a good outcome with previously prescribed antibiotic	0.87	-0.06	0.71	0.28	2.56	0.39
	Give antibiotics to poor patients who cannot afford doctor/hospital charges keeping in mind their cost-effectiveness	0.87	-1.24	0.89	0.10	8.37	0.11
Follow the drug act and do not dispense antimicrobials without prescription	Evaluate patients and dispense antimicrobials based on symptoms, and not their age or gender	0.96	0.21	0.68	0.31	2.12	0.47
	Give antibiotics if the patient has a good outcome with previously prescribed antibiotic	0.96	-0.06	0.73	0.26	2.80	0.35
	Give antibiotics to poor patients who cannot afford doctor/hospital charges keeping in mind their cost-effectiveness	0.96	-1.24	0.90	0.09	9.18	0.10
Do not dispense antimicrobials without prescription for a pediatric or geriatric patient with severe infection	Follow the drug act and do not dispense antimicrobials without prescription	0.36	0.96	0.35	0.64	0.54	1.83
	Evaluate patients and dispense antimicrobials based on symptoms, and not their age or gender	0.36	0.21	0.53	0.46	1.16	0.86
	Dispense antibiotics if the patient has a good outcome with previously prescribed antibiotic	0.36	-0.06	0.60	0.39	1.53	0.65
	Give antibiotics to poor patients who cannot afford doctor/hospital charges keeping in mind their cost-effectiveness	0.36	-1.24	0.83	0.16	5.01	0.19
Evaluate patients and dispense antimicrobials based on symptoms, and not their age or gender	Do not dispense antimicrobials and usually refer the patient to get a prescription from a doctor if the patent has a high fever, generalized malaise, fatigue as symptoms, sinusitis	0.21	0.87	0.34	0.65	0.51	1.93
	Give antibiotics if the patient has a good outcome with previously prescribed antibiotic	0.21	-0.06	0.56	0.43	1.32	0.75
	Dispense antibiotics to poor patients who can not afford doctor/hospital charges keeping in mind their cost-effectiveness	0.21	-1.24	0.81	0.18	4.31	0.23
Dispense antibiotics if the patient has a good outcome with previously prescribed antibiotic	Dispense antimicrobials when patient requests for it without a prescription	-0.06	-0.71	0.65	0.34	1.91	0.52
	Dispense antibiotics to poor patients who cannot afford doctor/hospital charges keeping in mind their cost-effectiveness	-0.06	-1.24	0.76	0.23	3.26	0.30
Dispense antibiotics to poor patients who cannot afford doctor/hospital charges keeping in mind their cost-effectiveness	Dispense antimicrobials if the patient has a mild fever	-1.24	-0.39	0.29	0.70	0.42	2.35
	Dispense antimicrobials when patient requests for it without a prescription	-1.24	-0.71	0.36	0.63	0.58	1.70

The all-level comparison report provides pairwise comparisons for the antimicrobial dispensing attribute variables. The first and second components of the comparison represent Compared 1 and Compared 2, which are the factor and levels, respectively. Utility 1 and Utility 2 are the estimated utilities for the antimicrobial dispensing attribute variables specified in the first and second components. Probability 1 is the predicted likelihood that the first component will be preferred over the second component for the specified antimicrobial dispensing attribute variables in the first component. Similarly, Probability 2 is the predicted likelihood that the second component will be preferred over the first component for the specified antimicrobial dispensing attribute variables in the second component. Additionally, Odds 1 is obtained by dividing Probability 1 by Probability 2, while Odds 2 is obtained by dividing Probability 2 by Probability 1.

On average, medicine sales outlet staff are more likely to refer patients with high fevers, malaise, fatigue, and sinusitis to a doctor for a prescription (odds ratio = 4.89; probability = 0.83, odds ratio = 1.66; probability = 0.62) compared to dispensing antimicrobials when a patient requests them without a prescription (odds ratio = 0.20; probability = 0.16) as well as not dispensing antimicrobials without a prescription for pediatric or geriatric patients with severe infections (odds ratio = 0.59; probability = 0.37). Following the drug act by not dispensing antimicrobials without a prescription has higher odds (odds ratio = 2.12; probability = 0.68) than evaluating patients based on symptoms (odds ratio = 0.47; probability = 0.31). Medicine sales outlet staff are more likely to refer patients with high fevers, malaise, fatigue, and sinusitis to a doctor for a prescription (odds ratio = 1.93; probability = 0.65) compared to evaluating patients based on symptoms (odds ratio = 0.51; probability = 0.34).

## Discussion

Antimicrobial drugs are widely used by patients worldwide. However, these drugs are easily available and can be obtained in developing and middle-income countries without a prescription from various medicine sales outlets (i.e., pharmacies and licensed drug stores) because the pharmaceutical industry needs proper regulation. This study assessed antimicrobial prescribing and dispensing practices in medicine sales outlets. The study provides quantitative evidence to enhance patient safety and care quality in Ghana and other developing and middle-income countries where antimicrobial drugs are easily accessible. The study found that most respondents were males aged 18–30 years. Most medicine sales outlet staff were pharmacists with 1–5 years of experience working in pharmacy stores. They had participated in several antimicrobial training programs organized by the pharmacy council and their facility manager or professional staff. These findings support earlier observations that personnel at the drug store gained their pharmaceutical knowledge through on-the-job training, conversations with pharmacists, advertisements, common knowledge about medicines in the community, client feedback, and presentations by drug representatives at association meetings [1].

A key finding of this study is that staffs at the medicine sales outlets, such as pharmacies and licensed drug stores, are highly concerned with following the drug act and not dispensing antimicrobials without a prescription. Other stronger preferences were observed for medicine sales outlet staff not dispensing antimicrobials and instead referring patients to a doctor if they have complications like high fever, fatigue, generalized malaise, or sinusitis, followed by not dispensing antimicrobials without a prescription for pediatric or geriatric patients with severe infection and evaluate patients based on their symptoms, not age or gender. However, medicine sellers tend not to dispense antibiotics if a patient only has a mild fever and requests antibiotics without a prescription.

Our results revealed that medicine sellers prioritize following laws and guidelines to control the use of antimicrobials and not dispensing them without a prescription. They also prioritize referring patients to a doctor for a prescription if they exhibit symptoms like high fever, fatigue, generalized malaise, or sinusitis. However, previous studies have shown conflicting results on antibiotic prescribing and dispensing rates, with some patients purchasing antibiotics without a prescription and personally requesting specific drugs without seeking advice from medicine outlet staff [1,2,36,37]. These contrasting findings highlight the need for evidence-based antimicrobial prescribing and dispensing practices at medicine sales outlets in Ghana and other developing and middle-income nations where antimicrobials are easily accessible. A national surveillance system is needed to monitor antimicrobial prescribing and dispensing practices, which would help policymakers to know the pattern of commonly used antimicrobials in the medicine sales outlets [1] . This would allow for the evidence-based selection of antimicrobials for treating infectious diseases [37] and contribute significantly to the fight against antimicrobial resistance [7].

This study further revealed that staff members at medicine outlets evaluate patients and dispense antimicrobials based on symptoms rather than age or gender. This result is in line with acceptable regulations [14] because pharmacists are authorized to prescribe certain drugs, such as Amoxicillin, Flucloxacillin, Norfloxacin + Tinidazole, Ciprofloxacin, Doxycycline, Tetracycline, Erythromycin, and Ampicillin without a doctor’s prescription while keeping accurate records of transactions [13]. However, the practice of prescribing and dispensing antimicrobials by unlicensed individuals in medicine sales outlets violates the Ghana National Action Plan on Antimicrobial Resistance. It can lead to the misuse of antimicrobials, resulting in community-wide antimicrobial resistance, unsuccessful treatments, and high healthcare costs for patients and the state [7,37].

Our results revealed that medicine sales outlet staff hesitated to provide antibiotics to patients with mild fevers and did not give out antimicrobials without a prescription. A previous study revealed that only 46% of pharmacists reported always giving antibiotics with a prescription. However, the most frequently prescribed antibiotics without a prescription were Amoxicillin, Metronidazole, and Cephalexin, accounting for 51% of cases [38]. It is essential to implement regulations for dispensing antimicrobial drugs, offer ongoing education, and provide training to medicine outlet staff, including pharmacists and pharmacy assistants, to address the issue of antimicrobial resistance. These can be achieved through organizations like the Pharmacy Council [15], pharmaceutical companies, the Ghana Health Service (GHS), and the Ministry of Health (MOH).

According to our findings, dispensing antimicrobials to patients with mild fevers is better than providing them to patients who request them without a prescription [3,7]. If a patient has a high fever, general malaise, fatigue, or sinusitis, referring them to a doctor for a prescription instead of dispensing antimicrobials is best. It is important to adhere to the drug act [14] and not give out antimicrobials without a prescription, especially for pediatric or geriatric patients with severe infections. Patients should be evaluated based on symptoms rather than age or gender when dispensing antimicrobials. Dispensing antimicrobials without a prescription should be avoided, and patients should be referred to a doctor for a prescription if necessary, as covered in the Health Professions Regulatory Act, 2013 (Act 857) [13].

The authors of this study acknowledge certain limitations. Firstly, since the research was conducted solely in Accra, it may not accurately represent how antimicrobials are typically dispensed in other parts of Ghana and Sub-Saharan Africa. However, the easy drug access in Accra may reflect dispensing practices in other areas. Secondly, the study only investigates specific factors related to antimicrobial dispensing practices and does not cover other important attributes such as the type of antimicrobials dispensed, record maintained on antimicrobials dispensed, laboratory/diagnostic test of microbes before prescription of antimicrobials, patients demand antibiotics without prescription or when they do not fully understand their condition, utilization of already prescribed antibiotics for the same infection or illness, the proportion of the nonprescription sale of antimicrobials for patients with particular symptoms, among others are not covered. This may lead to biased utility estimates. Future studies should include these questions to address excluded attribute biases. Additionally, the study relied on provider reports, and their integrity could not be verified. However, future studies should employ observational study or anonymously approach medicine sales outlets as "patients" to validate the results.

Although there are limitations, this study provides important data on the dispensing of antimicrobials in medicine sales outlets. This information is crucial for policymakers to improve patient safety and care quality in the pharmaceutical industry in Sub-Saharan Africa, particularly in Ghana, where the misuse of antimicrobials has become a pressing issue. This misuse can lead to the development of antimicrobial resistance, resulting in ineffective treatment of infectious diseases and high healthcare costs for patients and the state. This study is the first to utilize the best-worst scaling experiment approach to assess antimicrobial dispensing practices.

## Supporting information

S1 Data (XLSX)
